# Serum cold-inducible RNA-binding protein (CIRP) levels as a prognostic indicator in patients with acute ischemic stroke

**DOI:** 10.3389/fneur.2023.1211108

**Published:** 2023-07-14

**Authors:** Mingming Li, Min Yao, Kangmei Shao, Xueyang Shen, Yongnan Li, Zhaoming Ge

**Affiliations:** ^1^Department of Neurology, Lanzhou University Second Hospital, Lanzhou University, Lanzhou, China; ^2^Gansu Provincial Neurology Clinical Medical Research Center, Lanzhou University Second Hospital, Lanzhou, China; ^3^Expert Workstation of Academician Wang Longde, Lanzhou University Second Hospital, Lanzhou, China; ^4^Department of Cardiac Surgery, Lanzhou University Second Hospital, Lanzhou University, Lanzhou, China

**Keywords:** acute ischemic stroke (AIS), biomarker, cold-inducible RNA-binding protein (CIRP), inflammation, prognosis

## Abstract

**Background:**

Acute ischemic stroke (AIS) is the leading cause of morbidity and mortality among cerebrovascular diseases. While animal studies have suggested a correlation between cold-inducible RNA-binding protein (CIRP) serum levels and the severity and prognosis of cerebral infarction, there has been a lack of research exploring this association in humans with cerebral infarction.

**Materials and methods:**

A total of 148 patients diagnosed with AIS within 7 days from symptom onset were included in this study. Comprehensive information regarding the patients' basic demographics, medical history, clinical parameters, the severity of cerebral infarction, and serum CIRP levels was collected. Follow-up data were obtained through telephonic interviews or by reviewing clinical notes for 3 months after the patients were discharged to assess the functional outcomes of treatment.

**Results:**

The findings of this study demonstrated a significant increase in serum CIRP levels during the early stages of AIS, followed by a gradual decline after 3 days. Significant differences were observed in the serum CIRP levels between the 1-day group and the 4–7 day group (*P* < 0.0047), as well as between the 2–3 day group and the 4–7 day group (*P* < 0.0006). Moreover, a significant positive correlation was observed between the serum CIRP levels and the severity of cerebral infarction. Higher serum CIRP levels were associated with more severe National Institutes of Health Stroke Scale scores (*P* < 0.05) and larger cerebral infarction volumes (*P* < 0.05). Furthermore, patients with higher serum CIRP levels exhibited poorer modified Rankin scale scores (*P* < 0.05). These findings indicate that serum CIRP serves as an essential pro-inflammatory mediator and a valuable biomarker for assessing brain injury in patients with AIS.

**Conclusion:**

The findings of this study suggest an elevation in serum CIRP levels among patients with AIS. These levels are positively correlated with the severity of AIS and serve as indicators of a poor prognosis. Therefore, CIRP could serve as a target for early clinical intervention while managing AIS, and further research should explore serum CIRP levels as prognostic indicators in AIS.

## 1. Introduction

Stroke ranks as the second leading cause of death and the third leading cause of disability globally, with its incidence and associated burdens rapidly increasing in several countries (1). In China, stroke poses a significant burden, with estimated prevalence, incidence, and mortality rates of 2.6%, 505.2 per 100,000 person-years, and 343.4 per 100,000 person-years, respectively, among adults aged ≥40 years in 2020 (2). Ischemic strokes are the most common stroke type, significantly affecting patients' prognoses and quality of life. From 1990 to 2019, there was a 70.0% increase in global stroke cases, an 85.0% increase in the number of patients with stroke, a 43.0% increase in stroke-related deaths, and a 32.0% increase in stroke-induced disabilities (3). Ischemic strokes result in neuronal cell death and the release of damage-associated molecular patterns (DAMPs) that elicit localized inflammation in the affected brain region. In addition to the localized inflammation, mounting evidence suggests that poststroke inflammatory responses might involve the entire brain and persist over a prolonged period, exerting long-term effects on patients' neurological health (4). Therefore, DAMPs are currently under investigation as potential biomarkers for monitoring stroke progression and severity.

Extracellular cold-inducible RNA-binding protein (CIRP) is a DAMP molecule that plays a crucial role in triggering inflammatory responses. The 172-amino acid RNA chaperone (5) is expressed in response to various stress conditions, including hypothermia, hypoxia, ultraviolet radiation, glucose deficiency, and heat stress, indicating its role as a stress-reactive protein (6). Consequently, CIRP is likely expressed in various tissues and organs such as the testis, brain, lungs, kidneys, liver, stomach, bone marrow, and heart (7). Numerous studies have highlighted the significant involvement of CIRP in acute lung injury, acute pancreatitis, Alzheimer's disease, sepsis, lung cancer, endometrial cancer, and other diseases (8–10). During hemorrhagic shock and sepsis, inflammation triggers the translocation of CIRP from the nucleus to the cytosol, leading to its release into the extracellular fluid (11). Extracellular CIRP (eCIRP) interacts with Toll-like receptor 4, triggering receptor expressed on myeloid cells-1 (TREM-1), and interleukin (IL)-6 receptor, thus inducing immune-mediated inflammatory responses in macrophages, neutrophils, lymphocytes, and dendritic cells (12).

Previous studies have revealed an increase in eCIRP when tumor necrosis factor-α (TNF-α) expression is upregulated in mouse brains after middle cerebral artery occlusion (MCAO). This upregulation triggers inflammation and contributes to neuronal damage in cerebral ischemia (13). However, there is limited information on the role of CIRP in human stroke cases. Therefore, this study aimed to examine whether serum CIRP levels could serve as diagnostic and evaluative biomarkers for strokes in humans.

## 2. Materials and methods

### 2.1. Study approval

The institutional ethics committee approved the study protocol, and written informed consent was obtained from each patient. Patients diagnosed with AIS were recruited from the Department of Neurology at Lanzhou University Second Hospital. Human plasma samples were collected following a protocol approved by the ethics committee of the Lanzhou University Second Hospital (2021A-505).

The inclusion criteria for this study were as follows: (i) diagnosis of AIS based on the presence of acute neurological deficit and confirmation of cerebral infarction through computed tomography or magnetic resonance imaging (MRI); (ii) patients aged >18 years; (iii) admission to the hospital within 7 days of stroke occurrence; and (iv) no history of intravenous thrombolysis or prior intravascular treatment.

The exclusion criteria for this study were as follows: (i) evidence of hemorrhagic infarction; (ii) severe comorbidities, including tumors, gastrointestinal bleeding, cirrhosis, acute or chronic renal failure, diabetic ketoacidosis or hypertonic state, chronic liver disease, recent infectious diseases, a recent history of abdominal disease, recent history of abdominal surgery, history of unexplained weight loss, unknown etiology (according to the Trial of Org 10172 in Acute Stroke Treatment), and decreased life expectancy; and (iii) unstable condition.

### 2.2. Baseline clinical data collection

Patient demographic information, medical history, clinical characteristics, and cerebral infarction volume data were extracted from the hospital records. The severity of the stroke at admission and upon discharge was assessed using the National Institutes of Health Stroke Scale (NIHSS) and the modified Rankin scale (mRS).

### 2.3. Biochemical analyses

Blood samples were collected on the second day after hospitalization. Prior to use, reagents were returned to room temperature and thoroughly mixed. A microtitre plate was prepared with standard wells, test sample wells, and blank wells, each set up in duplicate. In the standard wells, 50 μl of serum was added, while 10 μl of the serum samples were diluted five times with 40 μl of sample diluent in the test sample wells. The blank wells received 50 μl of sample diluent. Except for the blank wells, 100 μl of enzyme-labeled reagent was added to each well. The enzyme-linked immunosorbent assay (ELISA) plates were sealed and incubated in a thermostat at 37°C for 90 min. After removing the sealing film, the liquid was discarded, and the plate was spun dry. Subsequently, the wells were washed by adding diluted washing solution, allowing it to stand for 30 s, and then discarding it. The process of washing the plates was repeated five times, followed by patting the liquid dry using absorbent paper. Color developers A and B, 50 μl of each, were added to the plate wells, thoroughly shaken, and mixed. The plate was then incubated in a dark constant temperature oven at 37°C for 15 min. After adding 50 μl of stop solution to each well, the reaction was terminated, resulting in the color change from blue to yellow. Within 15 min of stopping the color change, the blank well was zeroed, and the absorbance of each well was measured at a wavelength of 450 nm.

### 2.4. Statistical analyses

Numerical variables are presented as the mean ± standard deviation (SD) or median with an interquartile range. Categorical variables are presented as percentages and compared using the Student's *t*-test or Mann-Whitney U test as appropriate. The normality of the data was assessed. Spearman's correlation analyses were performed to examine the associations between serum CIRP levels and various variables. All analyses were conducted using GraphPad Prism 7. Two-sided probability values were reported, and statistical significance was set at *P* < 0.05.

## 3. Results

### 3.1. Baseline characteristics of the study population

**Figure 1:**
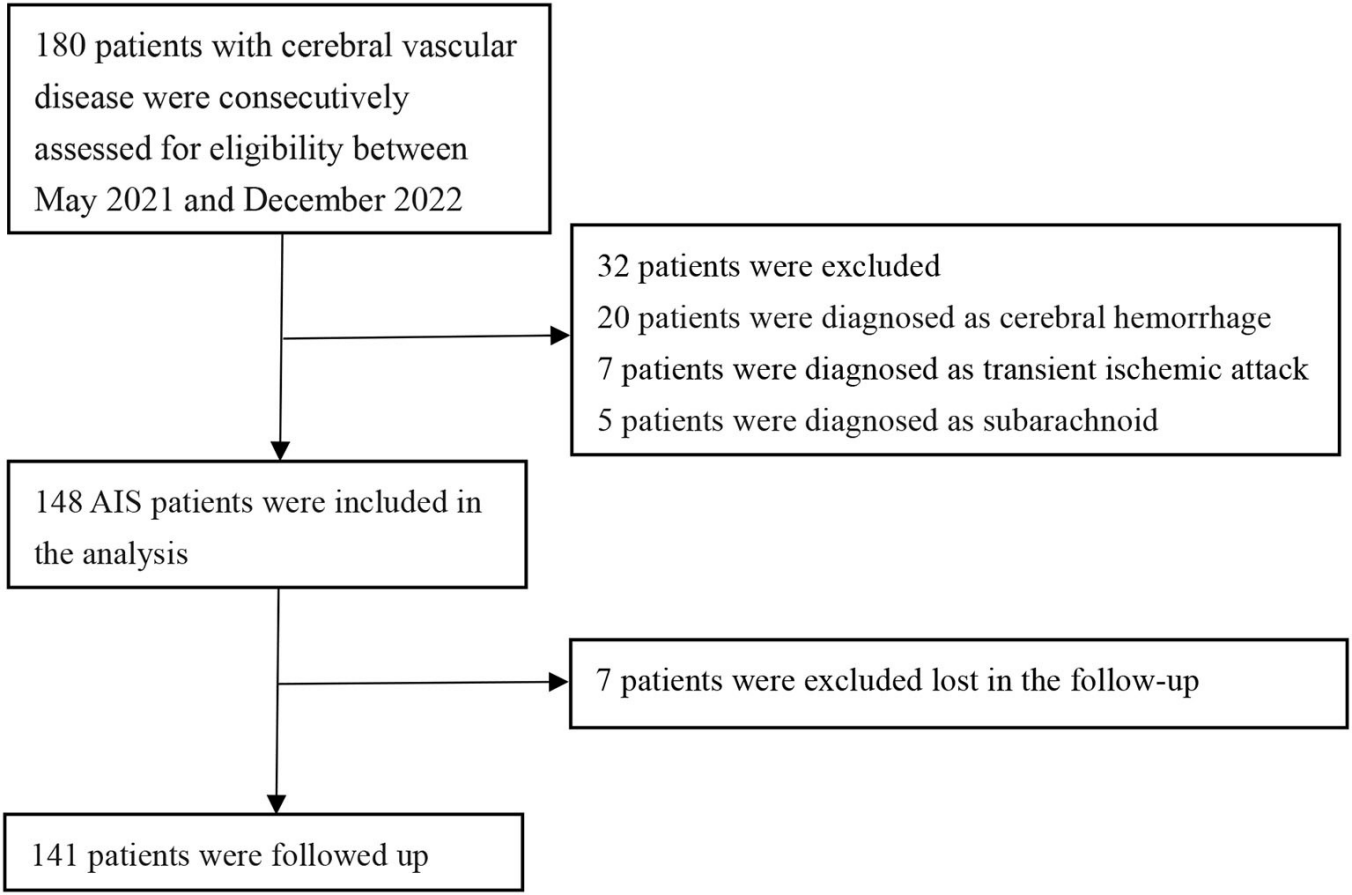
Flow diagram of the study.

A total of 148 patients were included in this retrospective review. These patients were admitted to Lanzhou University Second Hospital between May 2021 and December 2022 (Figure 1). Figure 1 illustrates the patients included and excluded in this study. The baseline characteristics of the participants are based on their incident acute ischemic stroke (AIS) status. The average age of the patient population in this study was 63.53 ± 10.69 years, with 109 (73.6%) patients being men. Among the participants, 28.86% (43 participants) were current or past smokers, 66.44% had hypertension, 30.87% had diabetes, and 12.08% had coronary heart disease (Table 1 and Supplementary Table 1). The average systolic blood pressure was 139.30 ± 21.65 mmHg, and the average diastolic blood pressure was 82.54 ± 14.00 mmHg. For the factors positively correlated with CIRP, further regression studies were conducted.

**Table 1:** Baseline characteristics of participants with acute ischemic stroke status.

**Variable**	**Value (*n* = 148)**
Age (years)	63.53 ± 10.69
Male, n/N (%)	109 (73%)
Body mass index, kg/m^2^	24.00 ± 3.10
Systolic blood pressure, mmHg	139.30 ± 21.65
Diastolic blood pressure, mmHg	82.54 ± 14.00
Smoke (*n*, %)	43 (28.86%)
Hypertension (*n*, %)	99 (66.44%)
Diabetes (*n*, %)	46 (30.87%)
Coronary heart disease (*n*, %)	18 (12.08%)
Glucose (mmol/L)	6.59 ± 3.40
Cystatin (mg/L)	0.95 ± 0.23
Total cholesterol, mmol/L	4.06 ± 1.03
Triglyceride, mmol/L	1.51 ± 0.76
HDL-C, mmol/L	0.99 ± 0.25
LDL-C, mmol/L	2.58 ± 0.76
Residual cholesterol, mmol/L	0.49 ± 0.56
WBC, 10^9^/L	6.75 ± 2.19
Neutrophils, 10^9^/L	4.31 ± 1.91
Lymphocyte, 10^9^/L	1.71 ± 0.63
Neutrophil/lymph, %	4.47 ± 18.63
Monocytes, 10^9^/L	0.54 ± 0.67
RBC, 10^12^/L	4.70 ± 0.57
Hemoglobin, g/L	144.17 ± 65.18
Platelet, 10^9^/L	198.19 ± 3.05
PDW, fl	13.97 ± 3.05
CRP, mg/L	6.03 ± 17.15

Data shown are *n* (%) or mean ± standard deviation.

HDL-C, high-density lipoprotein cholesterol; LDL, low-density lipoproteins cholesterol; WBC: white blood cell; RBC: red blood cells; PDW, platelet distribution width; CRP, C-reactive protein.

### 3.2. Serum CIRP levels in patients with AIS

**Figure 2:**
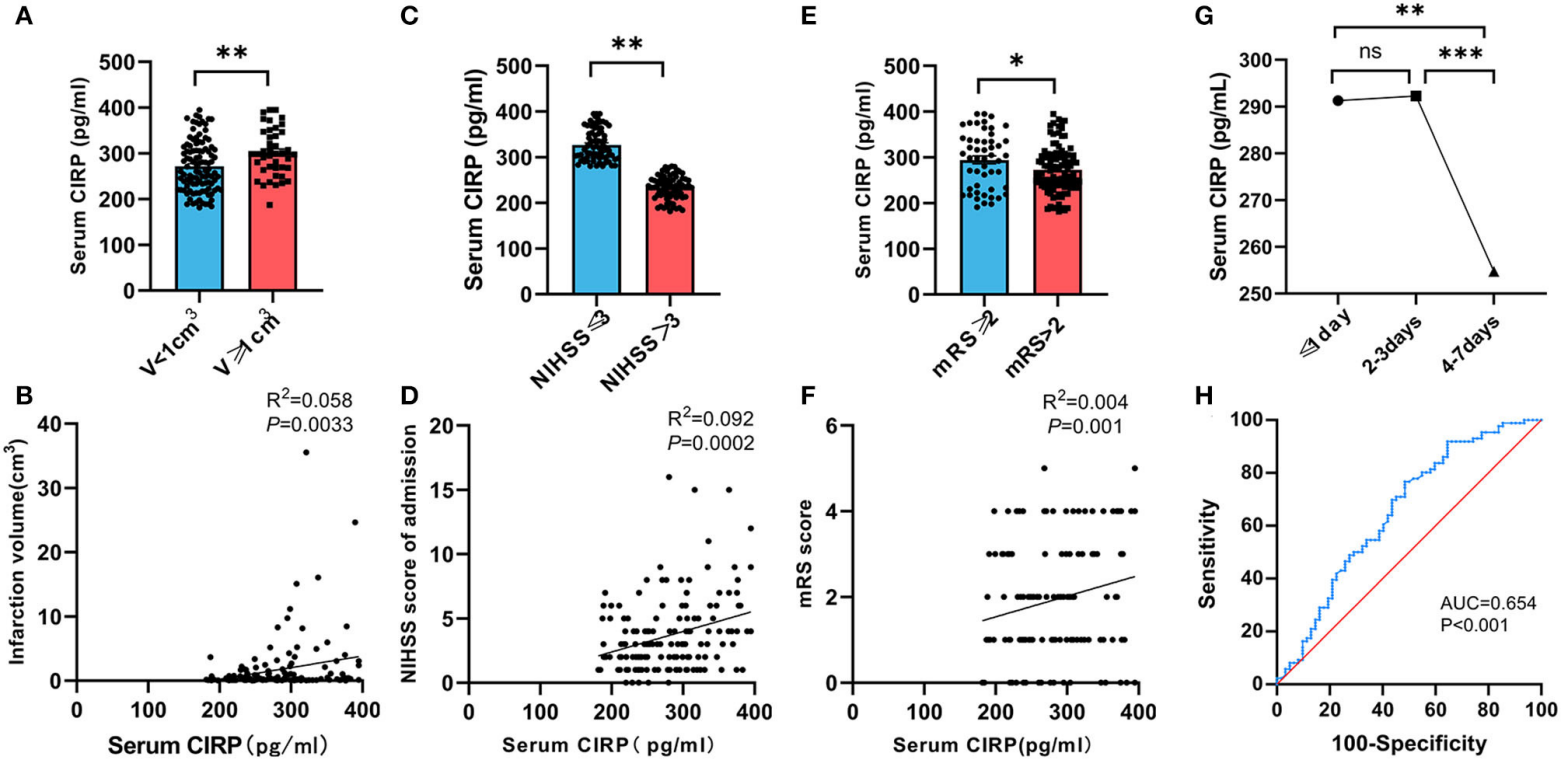
Relationship between serum CIRP level and severity of AIS. **(A)** Comparison of serum CIRP level in cerebral infarction volume (V) <1 cm^3^ group and ≥1 cm^3^ group. **(B)** Correlation between serum CIRP level and infarction volume calculated by Spearman's correlation analysis. **(C)** Comparison of serum CIRP level in NIHSS scores ≤ 3 group and >3 group. **(D)** Correlation between serum CIRP level and NIHSS scores by Spearman's correlation analysis. **(E)** Comparison of serum CIRP level in mRS scores ≤ 2 group and >2 group. **(F)** Correlation between serum CIRP level and mRS scores by Spearman's correlation analysis. **(G)** serum CIRP level in ≤ 1 day, 2–3 days, and 4–7 days; plotted are means ± standard error of the mean (SEM). **(H)** The ROC curve to evaluate the diagnostic value of CIRP for stroke. CIRP: cold-inducible RNA-binding protein. NHISS, National Institute of Health stroke scale; mRS, Modified Rankin Scale. ROC, receiver operating characteristic; CIRP, cold-inducible RNA-binding protein. *P < 0.05, **P < 0.01, ***P < 0.001.

The blood samples were collected *via* venipuncture from patients on an empty stomach in the morning following their admission. The serum CIRP levels were measured using ELISA. A positive correlation was observed between serum CIRP levels and the severity of brain infarction. The infarction volume of each patient was determined using the Pulicino formula based on data obtained from brain MRI, including T2 flair and diffusion-weighted imaging. The results demonstrated that patients with larger infarction volumes (V ≥ 1 cm^3^) had significantly higher serum CIRP levels compared to those with smaller infarction volumes (V <1 cm^3^) (*P* < 0.01). Correlation analysis revealed a positive correlation between serum CIRP levels and infarction volumes (*r* = 0.240, *P* < 0.01; Figures 2A, B).

The enrolled patients were divided into two groups based on their NHISS scores. The mild neurological deficit group (NIHSS ≤ 3) comprised 86 patients (58.11%), while the moderate and severe neurological deficit group (NIHSS >3) comprised 62 patients (41.89%). Comparative analysis between the groups revealed that patients with higher NHISS scores (298.39, 249.57–350.78) exhibited significantly higher serum CIRP levels compared to patients with lower NIHSS scores (267.34, 232.26–301.17) (*P* < 0.01). Additionally, a positive correlation was observed between serum CIRP levels and the severity of neurological impairment (*r* = 0.304, *P* < 0.01; Figures 2C, D).

All patients underwent a follow-up examination using a standard questionnaire survey 90 days after the onset of cerebral infarction. The follow-ups were conducted either in the outpatient department of the hospital or by telephone. Of the 148 patients included in this study, 7 patients were lost to follow-up. The short-term prognoses of the remaining 141 patients, evaluated using mRS, were used for the cohort study, and the patients were divided into two groups. The poor prognosis group comprised 21 patients (14.89%), while the good prognosis group comprised 120 patients (85.11%). Correlation analysis revealed a positive correlation between the serum CIRP levels and the mRS scores of these patients (*r* = 0.04, *P* < 0.01; Figures 2E, F).

Subsequently, the patient population included in this study was categorized into three groups based on the onset time of cerebral infarction: the 1-day group (*n* = 37), the 2–3 day group (*n* = 65), and the 4–7 day group (*n* = 46). The mean ± SD values of the serum CIRP levels were 291.27 ± 56.32 pg/ml, 292.25 ± 53.86 pg/ml, and 254.74 ± 51.42 pg/ml for the 1-day, 2–3 day, and 4–7 day groups, respectively. Additionally, it was observed that the serum CIRP levels of these patients peaked within 3 days of cerebral infarction and subsequently declined. No statistically significant differences were observed in the serum CIRP levels between the 1-day and 2–3 day groups (*P* < 0.883). However, significant differences were observed in the serum CIRP levels between the 1-day and 4–7 day groups (*P* < 0.0047) and between the 2–3 day and 4–7 day groups (*P* < 0.0006) (Figure 2G).

### 3.3. Comparison of the serum CIRP levels in patients with AIS

Serum CIRP levels were not normally distributed, as confirmed by the Kolmogorov–Smirnov normality test. Therefore, these levels were reported as median and range. Based on previous literature (20), the patient population in this study was divided into two groups using the median value (279.94 pg/ml) of the serum CIRP levels. These groups were classified as the low expression group (serum CIRP levels <279.94 pg/ml; 235.90 [215.93–251.63 pg/ml]) and the high expression group (serum CIRP levels >279.94 pg/ml; 317.94 [299.23–357.57 pg/ml]). The high expression group had a significantly higher frequency of coronary heart disease history (*P* < 0.012) and significantly higher serum total cholesterol (*P* = 0.025), low-density lipoprotein cholesterol (*P* = 0.034), remnant cholesterol (*P* < 0.029), and residual cholesterol (*P* < 0.029) levels than those in the low expression group (Table 2). Furthermore, the low expression group had significantly lower NIHSS scores (*P* < 0.013), mRS scores (*P* < 0.049), and lower infarct volumes in head MRIs (*P* < 0.046) compared with the high expression group (Table 3).

**Table 2:** Baseline characteristics of AIS patients between high levels of CIRP group and low levels of CIRP group.

**Variable**	**CIRP <279.94 pg/ml group (*n* = 74)**	**CIRP > 279.94 pg/ml group (*n* = 74)**	***P*-value**
Age (years)	62.93 ± 11.40	64.12 ± 10.05	0.502
Men, n/N (%)	54/74 (73%)	55/74 (74%)	0.852
Body mass index, kg/m^2^	23.60 ± 3.20	24.39 ± 2.98	0.122
Systolic blood pressure, mmHg	138.47 (126.25–146)	140.12 (125–154)	0.585
Diastolic blood pressure, mmHg	81.72 ± 13.86	83.36 ± 14.28	0.477
Smoke (*n*, %)	24 (32.4%)	19 (25.7%)	0.877
Hypertension (*n*, %)	48 (64.9%)	51 (68.9%)	0.602
Diabetes (*n*, %)	26 (35.1%)	20 (27%)	0.288
Coronary heart disease^*^ (*n*, %)	4 (5.4%)	14 (18.9%)	0.012
Glucose (mmol/L)	6.42 (4.43–7.41)	6.76 (4.64–7.32)	0.79
Cystatin (mg/L)	0.95 (0.82–1.06)	0.98 (0.86–1.08)	0.523
Total cholesterol, mmol/L ^*^	3.80 ± 1.13	4.20 ± 1.05	0.025
Triglyceride, mmol/L	1.47 (0.99–1.77)	1.56 (0.99–1.91)	0.72
HDL-C, mmol/L	1.00 (0.823–1.13)	0.98 (0.82–1.10)	0.506
LDL-C, mmol/L ^*^	2.45 ± 0.84	2.72 ± 0.67	0.034
Residual cholesterol, mmol/L ^*^	0.40 (0.17–0.56)	0.58 (0.26–0.65)	0.029
WBC, 109/L	6.61 (5.24–7.55)	6.90 (5.4–8.13)	0.539
Neutrophils, 109/L	4.22 (3.01–4.93)	4.40 (3.12–5.08)	0.484
Lymphocyte, 109/L	1.69 ± 0.55	1.73 ± 0.72	0.671
Neutrophil/lymph	2.82 (1.59–3.23)	6.12 (1.60–3.70)	0.672
Monocytes, 109/L	0.50 (0.37–0.60)	0.58 (0.38–0.54)	0.864
RBC, 1,012/L	4.71 ± 0.60	4.70 ± 0.56	0.867
Hemoglobin, g/L	143.77 (133–157)	144.57 (133–157.5)	0.683
Platelet, 109/L	196.54 (153–239.25)	199.84 (149.5–236.25)	0.778
PDW, fl	13.68 (11.98–16)	14.28 (11.5–16.3)	0.503
CRP, mg/L	5.58 (1.17–4.32)	7.20 (0.64–4.93)	0.726

Data shown are *n* (%) or mean ± standard deviation.

HDL-C, high-density lipoprotein cholesterol; LDL, low-density lipoprotein cholesterol; WBC, white blood cell; RBC, red blood cells; PDW, platelet distribution width; CRP, C-reactive protein.

^*^P < 0.05. ^**^P < 0.01.

**Table 3:** Disease severity and prognosis in AIS patients between high levels of CIRP group and low levels of CIRP group.

**Variable**	**CIRP <279.94 pg/ml group (*n* = 74)**	**CIRP > 279.94 pg/ml group (*n* = 74)**	***P*-value**
Cerebral infarction volume, kg3^*^	0.81 (0.12–0.92)	2.57 (0.12–2.15)	0.046
Time of cerebral infarction^*^	3.23 (2–5)	2.58 (1–3)	0.047
CIRP, pg/ml^**^	233.44 (215.93–251.63)	327.26 (299.23–357.57)	0.000
NIHSS score^*^	2.97 (1–4)	4.38 (2–6)	0.013
Admission mRS score^*^	1.7 (1–2.25)	2.15 (1–3.25)	0.049
mRS score at 90 days^*^	0.77 (0.00–1.00)	1.19 (0.00–2.00)	0.024

Data shown are *n* (%) or mean ± standard deviation.

CIRP, cold-inducible RNA-binding protein; NIHSS, National Institutes of Health Stroke Scale; mRS, modified Rankin Scale.

^*^P < 0.05. ^**^P < 0.01.

The regression model showed that BMI, history of coronary heart disease, LDL-C, and RC had a significant impact on serum CIRP levels (Supplementary Table 2). As shown in Supplementary Table 2, higher CIRP (OR = 1.011, 95% CI = 1.004–1.017, *P* < 0.01) was a risk factor for stroke. Furthermore, according to the ROC curve in Figure 2, the AUC of CIRP for predicting stroke was 0.654, 95% CI = 0.562–0.746, *P* < 0.001. Besides, the optimal cutoff value of CIRP for stroke was 303.24 pg/ml, with a specificity of 76.7% and a sensitivity of 51.6% (Figure 2H).

## 4. Discussion

In this study, an initial increase in serum CIRP levels was observed during the early stages of AIS, followed by a gradual decrease after 3 days. Additionally, a significant and positive correlation was observed between serum CIRP levels and the severity of cerebral infarction, as evidenced by higher NIHSS scores and larger cerebral infarction volumes. Patients with higher serum CIRP levels also exhibited poorer mRS scores. These findings suggest that serum CIRP levels could serve as a valuable indicator of pro-inflammatory events and might serve as an important biomarker for assessing brain injury in patients with AIS.

AIS is a medical emergency caused by decreased blood flow to the brain, resulting in cellular damage (14). This damage triggers the release of DAMPs from necrotic neurons, exacerbating inflammatory responses, and thereby worsening the prognosis of AIS. The DAMPs involved in these processes include CIRP, high mobility group box 1, histones, and other proteins (15). CIRP, an RNA chaperone responsive to stress conditions, such as hypoxia and mild hypothermia (16), is widely expressed in various cell types and shares conserved RNA recognition sequences with closely related proteins (17). It plays a significant role in multiple cellular signaling pathways as a stress response protein (18). Notably, during cerebral infarction, eCIRP induces the release of inflammatory mediators and contributes to neuronal damage (11). Ischemia triggers the transfer of CIRP from the nucleus to the cytoplasm, followed by its release into the bloodstream (19). A study by Denning et al. identified eCIRP as a biologically active endogenous ligand of TREM-1, a mediator of inflammation during sepsis (20). Furthermore, a clinical study demonstrated that serum CIRP levels could serve as an independent prognostic biomarker, enhancing the predictive value of the Global Registry of Acute Coronary Events score for assessing the prognosis in patients with acute coronary syndrome (21). In a separate study, Zhou et al. revealed elevated serum CIRP levels in mice with upregulated TNF-α expression following MCAO, thereby stimulating inflammation and causing neuronal damage in cerebral ischemia (13). However, the serum CIRP levels in individuals with AIS have not been explored thus far. In this study, it was found that serum CIRP levels are elevated in patients with AIS and that these levels are positively correlated with the severity and prognosis of cerebral infarction.

The NIHSS score is a widely accepted measure for evaluating a patient's level of consciousness and neurological deficits (22, 23). Similarly, the mRS is commonly employed to assess global disability and serves as an endpoint measure in randomized clinical trials, particularly for patients who have experienced a stroke (24). The present study reported that patients with high serum CIRP levels had higher NIHSS and mRS scores, indicating poor neurological function and outcomes.

This study has certain limitations. First, it was conducted in a single center, which may limit the generalizability of the findings. Additionally, the sample size was relatively small. Furthermore, the study excluded patients who received mechanical or intravenous thrombolysis, which may limit the applicability of the findings to the broader population of patients with AIS. Moreover, the follow-up period was limited to 3 months. Therefore, future research should involve multi-center studies with larger sample sizes and longer follow-up durations to validate the findings of this study. Additionally, direct comparisons between CIRP and established biomarkers, such as C-reactive protein, IL-6, and TNF-α, should be conducted to assess their predictive value, sensitivity, and specificity for stroke outcomes.

## 5. Conclusion

This study indicates that serum CIRP levels are elevated in patients with AIS. Additionally, it was observed that serum CIRP levels were positively correlated with the severity of AIS and poor prognosis. These findings indicate that CIRP could serve as a target for early clinical intervention in AIS management. Moreover, the significance of serum CIRP levels as a biomarker for predicting AIS prognosis warrants further investigation.

## Data availability statement

The original contributions presented in the study are included in the article/Supplementary material, further inquiries can be directed to the corresponding authors.

## Ethics statement

Human plasma samples were collected under a protocol approved by the Ethics Committee of the Lanzhou University Second Hospital (2021A-505). The patients/participants provided their written informed consent to participate in this study.

## Author contributions

ZG, YL, and ML contributed to the conception and design of the study. MY organized the database. KS performed the statistical analysis. XS wrote the manuscript. All authors contributed to the manuscript revision, and read and approved the submitted version.

## References

[1] Owolabi MO Thrift AG Mahal A Ishida M Martins S Johnson WD. Primary stroke prevention worldwide: translating evidence into action. The Lancet Public Health. (2022) 7:e74–85. 10.1016/S2468-2667(21)00230-9 34756176 PMC8727355

[2] Tu W-J Zhao Z Yin P Cao L Zeng J Chen H. Estimated burden of stroke in China in 2020. JAMA Netw Open. (2023) 6:e231455. 10.1001/jamanetworkopen.2023.1455 36862407 PMC9982699

[3] Global regional and and national burden of stroke and its risk factors 1990–2019: 1990–2019: a systematic analysis for the Global Burden of Disease Study 2019. The Lancet. (2021) 20:795–820. 10.1016/S1474-4422(21)00252-0 34487721 PMC8443449

[4] Sakai S Shichita T. Role of alarmins in poststroke inflammation and neuronal repair. Seminars In Immunopathology. (2022) 10:5. 10.1007/s00281-022-00961-5 36161515

[5] Lujan DA Ochoa JL Hartley RS. Cold-inducible RNA binding protein in cancer and inflammation. Wiley Interdiscip Rev RNA. (2018) 9:1462. 10.1002/wrna.1462 29322631 PMC5886743

[6] Zhong P Peng J Yuan M Kong B Huang H. Cold-inducible RNA-binding protein (CIRP) in inflammatory diseases: molecular insights of its associated signalling pathways. Scand J Immunol. (2021) 93:e12949. 10.1111/sji.12949 32738154

[7] Zhong P Peng J Bian Z Huang H. The Role of Cold Inducible RNA-Binding Protein in Cardiac Physiology and Diseases. Front Pharmacol. (2021) 12:610792. 10.3389/fphar.2021.610792 33716740 PMC7943917

[8] Zhong P Zhou M Zhang J Peng J Zeng G Huang H. The role of Cold-Inducible RNA-binding protein in respiratory diseases. J Cell Mol Med. (2022) 26:957–65. 10.1111/jcmm.17142 34953031 PMC8831972

[9] Sharma A Brenner M Wang P. Potential Role of Extracellular CIRP in Alcohol-Induced Alzheimer's Disease. Molecular Neurobiology. (2020) 57:5000–10. 10.1007/s12035-020-02075-1 32827106 PMC7544648

[10] Zhou M Aziz M Yen H-T Ma G Murao A Wang P. Extracellular CIRP dysregulates macrophage bacterial phagocytosis in sepsis. Cellular & Mo-lecular Immunology. (2023) 20(1): 80-93. 10.1038/s41423-022-00961-3 36471113 PMC9794804

[11] Aziz M Brenner M Wang P. Extracellular CIRP (eCIRP) and inflammation. J Leukoc Biol. (2019) 106:133–46. 10.1002/JLB.3MIR1118-443R 30645013 PMC6597266

[12] Han J Zhang Y Ge P. Exosome-derived CIRP: An amplifier of inflammatory diseases. Front Immunol. (2023) 14:1066721. 10.3389/fimmu.2023.1066721 36865547 PMC9971932

[13] Zhou M Yang WL Ji Y Qiang X Wang P. Cold-inducible RNA-binding protein mediates neuroinflammation in cerebral ischemia. Biochimica Et Biophysica Acta. (2014) 1840:2253–61. 10.1016/j.bbagen.2014.02.027 24613680 PMC4061249

[14] Walter K. What is acute ischemic stroke? JAMA. (2022) 327:885. 10.1001/jama.2022.1420 35230392

[15] Maehara N Taniguchi K Okuno A Ando H Hirota A Li Z. AIM/CD5L attenuates DAMPs in the injured brain and thereby ameliorates ischemic stroke. Cell Rep. (2021) 36:109693. 10.1016/j.celrep.2021.109693 34525359

[16] Chen L Tian Q Wang W. Association between CIRP expression and hypoxic-ischemic brain injury in neonatal rats. Experi-mental and therapeutic medicine. (2019) 18:1515–20. 10.3892/etm.2019.7767 31410103 PMC6676150

[17] Logan SM Storey KB. Cold-inducible RNA-binding protein Cirp, but not Rbm3, may regulate transcript processing and protection in tissues of the hibernating ground squirrel. Cell Stress and Chaperones. (2020) 25(6): 857-868. 10.1007/s12192-020-01110-3 32307648 PMC7591650

[18] Denning N-L Aziz M Gurien SD Wang P. DAMPs and NETs in Sepsis. Front Immunol. (2019) 10:2536. 10.3389/fimmu.2019.02536 31736963 PMC6831555

[19] Liao Y Tong L Tang L Wu S. The Role of Cold-inducible RNA binding protein (CIRP) in Cell Stress Response. International Journal of Cancer. (2017) 141:833. 10.1002/ijc.30833 28608439

[20] Denning N-L Aziz M Murao A Gurien SD Ochani M Prince JM. Extracellular CIRP as an endogenous TREM-1 ligand to fuel inflammation in sepsis. JCI insight. (2020) 5:4172. 10.1172/jci.insight.134172 32027618 PMC7141396

[21] Ren X Xie H Zhang J Jin X Cui L Chen L. Prognostic Value of Plasma Cold-Inducible RNA-Binding Protein in Patients with Acute Coronary Syndrome. Dis Markers. (2022) 2022:6119601. 10.1155/2022/6119601 35531472 PMC9068342

[22] Yang Y Huang S Jia Y Song G Ye X Lu K. A 6-month prognostic nomogram incorporating hemoglobin level for intracerebral hemorrhage in younger adults. BMC Neurol. (2023) 23:6. 10.1186/s12883-022-03039-9 36609246 PMC9817395

[23] Wang Y Zhang Y Huang J Chen X Gu X Wang Y. Increase of circulating miR-223 and insulin-like growth factor-1 is associated with the pathogenesis of acute ischemic stroke in patients. BMC Neurol. (2014) 14:77. 10.1186/1471-2377-14-77 24708646 PMC4234389

[24] Banks JL Marotta CA. Outcomes validity and reliability of the modified Rankin scale: implications for stroke clinical trials: a literature review and synthesis. Stroke. (2007) 38:1091–6. 10.1161/01.STR.0000258355.23810.c6 17272767

