# Corrigendum: Serum cold-inducible RNA-binding protein (CIRP) levels as a prognostic indicator in patients with acute ischemic stroke

**DOI:** 10.3389/fneur.2023.1290135

**Published:** 2023-10-03

**Authors:** Mingming Li, Min Yao, Kangmei Shao, Xueyang Shen, Yongnan Li, Zhaoming Ge

**Affiliations:** ^1^Department of Neurology, Lanzhou University Second Hospital, Lanzhou University, Lanzhou, China; ^2^Gansu Provincial Neurology Clinical Medical Research Center, Lanzhou University Second Hospital, Lanzhou, China; ^3^Expert Workstation of Academician Wang Longde, Lanzhou University Second Hospital, Lanzhou, China; ^4^Department of Cardiac Surgery, Lanzhou University Second Hospital, Lanzhou University, Lanzhou, China

**Keywords:** acute ischemic stroke (AIS), biomarker, cold-inducible RNA-binding protein (CIRP), inflammation, prognosis

In the published article, there was an error in the author list as published. Author Zhaoming Ge was listed in the fifth position, but should have been the last author.

The authors apologize for this error and state that this does not change the scientific conclusions of the article in any way. The original article has been updated.

