# Supplementary material for: Serum cold-inducible RNA-binding protein (CIRP) levels as a prognostic indicator in patients with acute ischemic stroke
Source: Front Neurol. 2023 Jul 14;14:1211108. doi: 10.3389/fneur.2023.1211108 (PMC10381024; doi:10.3389/fneur.2023.1211108)
Supplement: Supplementary file 1 [file Data_Sheet_1.docx]

**Supplemental Table 1.** baseline characteristics of participants according to gender

| **Variable** | **Male(n=109)** | **Female(39)** | **Value (n = 148)** |
| --- | --- | --- | --- |
| Age (years) | 61.91 ± 10.52 | 68.05 ± 10.10 | 63.53 ± 10.69 |
| Body mass index, kg/m^2^ | 24.01 ± 3.09 | 23.96 ± 3.19 | 24.00 ± 3.10 |
| Systolic blood pressure, mmHg | 137.45 ± 19.50 | 144.46 ± 26.61 | 139.30 ± 21.65 |
| Diastolic blood pressure, mmHg | 83.00 ± 13.91 | 81.26 ± 14.56 | 82.54 ± 14.00 |
| Smoke (n, %) | 42 (38.53%) | 1 (3%) | 43 (28.86%) |
| Hypertension (n, %) | 65 (59.63%) | 34 (87.18%) | 99 (66.44%) |
| Diabetes (n, %) | 35 (32.11%) | 11 (28.21%) | 46 (30.87%) |
| Coronary heart disease (n, %) | 10 (9.17%) | 8 (20.51%) | 18 (12.08%) |
| Glucose (mmol/L) | 6.52 ± 3.61 | 6.79 ± 2.81 | 6.59 ± 3.40 |
| Cystatin (mg/L) | 1.01 ± 0.24 | 0.91 ± 0.18 | 0.95 ± 0.23 |
| Total cholesterol, mmol/L | 3.98 ± 1.09 | 4.29 ± 0.83 | 4.06 ± 1.03 |
| Triglyceride, mmol/L | 1.46 ± 0.68 | 1.66 ± 0.94 | 1.51 ± 0.76 |
| HDL-C, mmol/L | 0.95 ± 0.24 | 1.12 ± 0.25 | 0.99 ± 0.25 |
| LDL-C, mmol/L | 2.53 ± 0.80 | 2.72 ± 0.66 | 2.58 ± 0.76 |
| Residual cholesterol, mmol/L | 0.50 ± 0.62 | 0.45 ± 0.33 | 0.49 ± 0.56 |
| WBC, 10^9^/L | 6.98 ± 2.21 | 6.12 ± 2.05 | 6.75 ± 2.19 |
| Neutrophils, 10^9^/L | 4.46 ± 1.93 | 3.86 ± 1.83 | 4.31 ± 1.91 |
| Lymphocyte, 10^9^/L | 1.71 ± 0.65 | 1.70 ± 0.60 | 1.71 ± 0.63 |
| Neutrophil/lymph, % | 5.06 ± 21.67 | 2.82 ± 3.65 | 4.47 ± 18.63 |
| Monocytes, 10^9^/L | 0.58 ± 0.77 | 0.42 ± 0.17 | 0.54 ± 0.67 |
| RBC, 10^12^/L | 4.78 ± 0.54 | 4.48 ± 0.62 | 4.70 ± 0.57 |
| Hemoglobin, g/L | 147.53 ± 23.96 | 134.77 ± 17.56 | 144.17 ± 65.18 |
| Platelet, 10^9^/L | 195.96 ± 62.03 | 204.41 ± 74.54 | 198.19 ± 3.05 |
| PDW, fl | 13.89 ± 2.96 | 14.21 ± 3.36 | 13.97 ± 3.05 |
| CRP, mg/L | 7.16 ± 19.64 | 4.25 ± 6.44 | 6.03 ± 17.15 |

Data shown are n (%) or mean ± standard deviation. HDL-C: High density lipoprotein cholesterol; LDL: Low density lipoproteins cholesterol; WBC: white blood cell; RBC: Red blood cells; PDW: platelet distribution width; CRP: C-reactiveprotein.

**Supplemental Table 2.** Factors contributed to moderate and severe stroke by the multivariate analysis.

| **Variables** | **OR** | **95%CI** | ***p*-value** |
| --- | --- | --- | --- |
| Age | 1.012 | 0.977-1.047 | 0.523 |
| Gender | 0.753 | 0.315-1.802 | 0.524 |
| CIRP | 1.011 | 1.004-1.017 | 0.002 |
| Coronary heart disease | 1.277 | 0.419-3.895 | 0.668 |
| LDL-C | 1.373 | 0.861-2.191 | 0.183 |
| Residual cholesterol | 0.743 | 0.376-1.465 | 0.391 |

CIRP, cold-inducible RNA-binding protein ; LDL-C, Low density lipoproteins cholesterol; OR, odds ratio; 95%CI, 95% confidence interval.
